# The Development of Healthcare Jobs in the COVID-Pandemic—A New Economic Market

**DOI:** 10.3389/fpubh.2022.848636

**Published:** 2022-04-04

**Authors:** Marta Bachmann, Bassey Enodien, Daniel M. Frey, Stephanie Taha-Mehlitz, Anas Taha

**Affiliations:** ^1^Department of Surgery, Wetzikon Hospital, Wetzikon, Switzerland; ^2^Clarunis, Department of Visceral Surgery, University Centre for Gastrointestinal and Liver Diseases, St. Clara Hospital and University Hospital Basel, Basel, Switzerland; ^3^Department of Biomedical Engineering, Faculty of Medicine, University of Basel, Allschwil, Switzerland

**Keywords:** healthcare jobs, COVID-19 pandemic, economic market, scoping review, jobs

## Abstract

**Background:**

The COVID-19 pandemic commenced in China and has caused the death of numerous people globally. Despite the adverse effects, the outbreak has created room for job opportunities in healthcare, particularly the pharmaceutical domain. The main goal of this study is to examine how the current pandemic has triggered job creation in the healthcare domain and created a new economic market.

**Methods:**

The study used the Preferred Reporting Items for Systematic and Meta-analyses for Scoping Review (PRISMA-ScR) to structure the manuscript and the subheadings to use. The source used to gather data is the PubMed database.

**Results:**

The study exclusively included fourteen articles, five of which focused on the pharmaceutical sector, three focused on vaccine sales, three on vaccination centers, and three on testing centers.

**Conclusion:**

The COVID-19 pandemic has created job opportunities in the healthcare sector. Most jobs are in the pharmaceutical sector, vaccination, and testing centers. However, more comprehensive research on the topic is necessary to gather conclusive outcomes on whether these jobs will be relevant after the pandemic.

## Introduction

### Rationale

The COVID-19 pandemic is a global virus outbreak that first appeared in Wuhan, China, after detecting numerous pneumonia cases at the end of 2019 (1). The virus has since spread and caused countless negative implications. The main challenge caused by the pandemic is the loss of lives, which has been significant globally. The healthcare domain has also felt the impacts of the pandemic, whereby there has been a substantial disruption of hospital services. The leading cause of these complications is the need to attend to emergency cases of the pandemic. Further, the pandemic has triggered vast unemployment rates among non-emergency healthcare workers in the domain due to the suspension of such services to contain the virus (2). Nevertheless, the pandemic has had some positive influences, particularly in healthcare. One of the possible impacts of the pandemic is the employment opportunities that it has created. The employment opportunities created are primarily in the pharmaceutical industry since currently, the only remedy to the disease is getting vaccinated. Using the scoping review approach to gather information on the jobs available in the healthcare domain as an outcome of the pandemic is appropriate for two reasons. First, the method will guarantee a descriptive evaluation of the available data on the topic under discussion. Second, the approach will ensure the transparency of the data collected, hence minimizing instances of bias.

### Objectives

The research question for this study is how has the COVID-19 pandemic created a new healthcare environment *via* job generation? Thus, the study's main aim is to evaluate how the current pandemic has triggered job creation in the healthcare domain and created a new economic market. The three research objectives outlined below will assist in exploring the question and aim further and responding to it. They include:

- To evaluate how the pandemic created jobs in the pharmaceutical industry.
- To assess how the pandemic created employment in the vaccine market and vaccination centers.
- To examine how the outbreak of COVID-19 triggered job creation in testing centers.

## Methods

### Protocol and Registration

The protocol developed for this research was the Preferred Reporting Items for Systematic and Meta-analyses for Scoping Review (PRISMA-ScR). The approach consists of 20 compulsory items and two optional variables that researchers conducting a scoping review must include in their manuscript. This method is appropriate for this study as it will permit the readers to evaluate the benefits and limitations of the research and allow the duplication of the review approaches integrated by other researchers studying the topic.

### Eligibility Criteria

The eligibility criteria for the articles used in this study significantly depended on several factors. Firstly, the authors of this manuscript selected the writings most relevant for the study based on the date of publication. Since COVID-19 is a recent occurrence that commenced in late 2019, the researchers exclusively include articles published between 2020 and 2021. These articles had a high chance of containing relevant information. Another criterion used to judge the publications included in the study is language. The researchers ensured that all the articles included were in English to prevent instances where they spent significant time translating articles released in foreign languages. Thirdly, the authors examined the papers based on the content. This factor was essential to guarantee that all the considered publications contained relevant information to the topic under discussion.

### Information Sources

Identifying the relevant documents for this study entailed a comprehensive literature search. The authors searched databases like PubMed and search engines like Google Scholar. The literature search explicitly searched for journals published in 2020 to the present date. The literature search occurred on December 14, 2021, and a qualified librarian in the team spearheaded the process and produced the search strategies to use. The other part of this process entailed the collaboration of all the authors to refine the search strategies integrated *via* a group discussion. Also, all the authors scanned the references found in relevant documents to gather additional journals to use. Other than that, the researchers hand-searched essential journals, mainly those found in the Google Scholar search engine, to acquire more information on the topic.

### Search

The search process adopted into this study entailed keying in the key terms on the PubMed database and the Google Scholar search engine. On the PubMed database, the researchers used words such as “the COVID-pandemic,” “vaccination jobs,” “pharmaceutical industry,” and “testing jobs.” The filters used here included “best match” and “one year”. On the Google Scholar search engine, the researchers used phrases like “the development of vaccine jobs after the pandemic,” “job creation in healthcare during the pandemic,” and “the future of the vaccine industry after COVID-19”. The filters used on the search engine were “since 2020” and “review articles”. The author who drafted the search strategy executed it. All the other authors peer-reviewed the search strategy to test its validity and reliability during the process. The researchers used the Peer Review of Electronic Search Strategies (PRESS) checklist to engage in this procedure. Through the approach, the authors proved that the sources gathered were acquired after an accurate translation of the study question.

### Selection of Evidence Sources

The choosing of evidence sources for use in this study involved the involvement of all the authors in a thorough screening process. All the authors engaged in the development of this manuscript screened all the articles all the acquired sources. They evaluated the subjects addressed by the article ad whether the abstracts and the full-text documents matched. This procedure prevented instances where an abstract lacked a complete text, thereby making it challenging to draw conclusive results of the articles. Before beginning the screening process, the authors examined the duplicate documents present and eliminated them to reduce the screening workload. The team resolved discrepancies mainly when authors disagreed on the eligibility of sources selected by reviewing the texts again to reach a non-biased consensus.

### Data Charting

Two authors engaged in the study developed a data charting form that comprised all the factors to look out for when gathering data. The form highlighted variables such as COVID-19 impacts and the opportunities presented by the pandemic in the medical domain. The two researchers mentioned above charted the information independently, after which they discussed the gathered results. This process continued where the authors updated the form iteratively. The authors resolved any misunderstandings and inconsistencies by involving the team, whereby everyone reviewed the data once more and established a consensus on the matter.

### Data Items

The process of extracting data depended on multiple features. Firstly, the researchers considered the contextual factors like the industry addressed by the articles. Secondly, the authors extracted articles based on their topic and whether they addressed the main research question of this study.

### Synthesis of Results

For this study, the researchers grouped the articles based on the main topic of study. The four categories identified were the pharmaceutical industry, vaccine sales, vaccination, and testing centers.

## Results

### Selection of Evidence Sources

**Figure 1:**
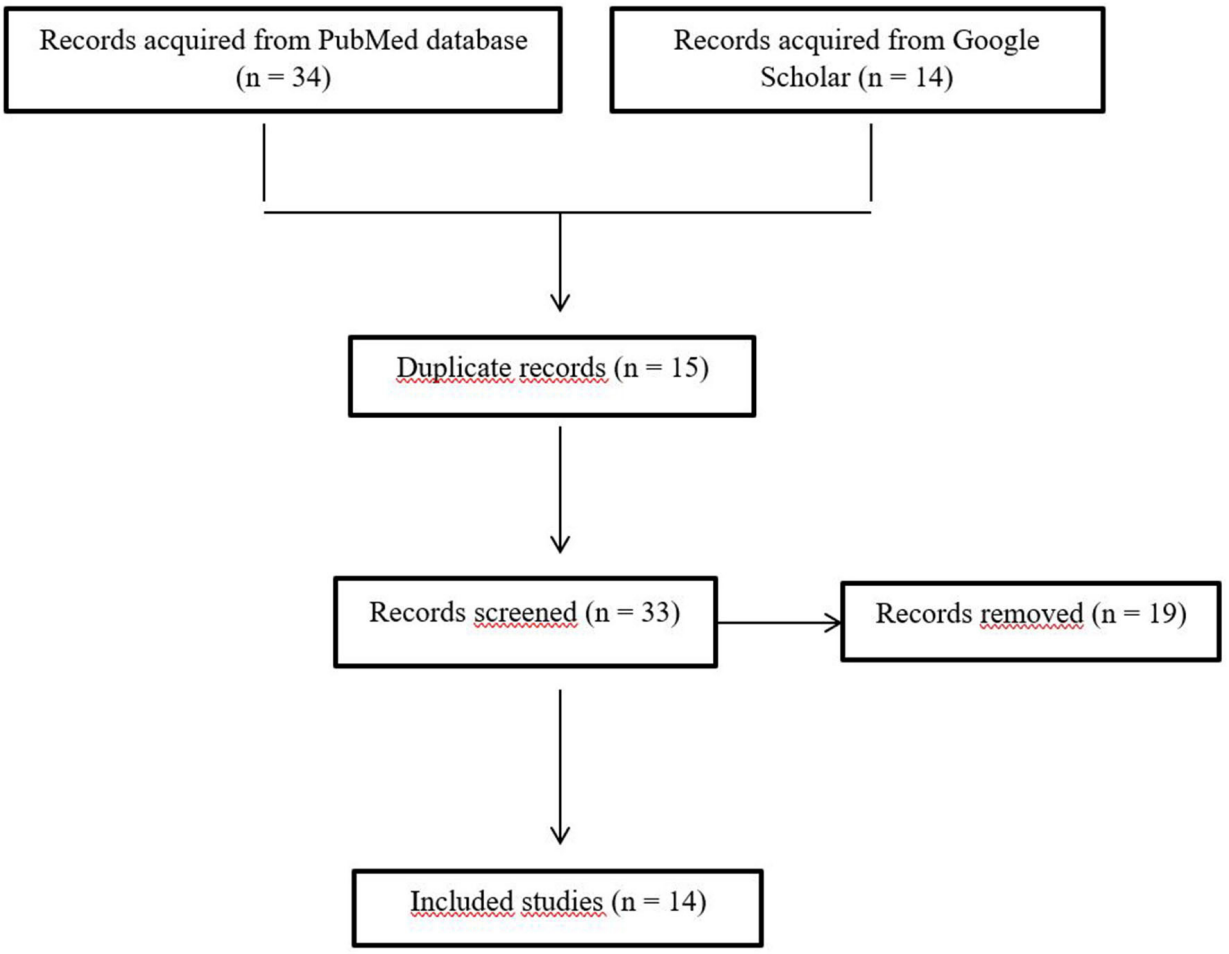
Inclusion and exclusion criteria for this study.

The search results on the PubMed database yielded 34 results, while Google Scholar produced 14 results. The entire inclusion and exclusion criteria adopted for this study are as evident in Figure 1.

### Results Synthesis

Out of the 14 included articles, five (35.8%) focused on the pharmaceutical industry, three (21.4%) on vaccine sales, three (21.4%) on vaccination centers, and three (21.4%) on testing centers as displayed in Table 1.

**Table 1:** Main themes of included studies.

**References**	**Primary theme**
**Pharmaceutical industry**	
Miglierini (3)	Pharmacies will offer long term employment opportunities in the future.
Morriss (4)	Many applicants seeking to enter the pharmaceutical industry since lockdown.
Singh (5)	Demand for medicine will create more job opportunities.
Akande-Sholabi and Adebisi (6)	Developing countries should produce their medicine domestically.
Ayati et al. (7)	Long-term impacts of COVID-19 include the pharmaceutical industry becoming self-sufficient.
**Vaccine sales**	
Zeitouny et al. (8)	Demand for vaccines was higher in low- and middle-income states than high-income countries during the first wave of the pandemic.
Bown and Bollyky (9)	Pfizer, Moderna, AstraZeneca, and Johnson & Johnson are the main produces of COVID-19 vaccines.
Oxfam International (10)	Pfizer, BioNTech, and Moderna make profits of one thousand dollars per second.
**Vaccination centers**	
Paudyal et al. (11)	The European continent has employed pharmacists to develop vaccination programs in vaccination centers.
Sahin and Sahin (12)	Vaccination centers present opportunities for physicians to identify other problems patients are encountering due to the pandemic.
Rubin (13)	The future of vaccination jobs is uncertain.
**Testing centers**	
Flynn et al. (14)	COVID-19 testing requires the contribution of healthy healthcare workers.
Fragala et al. (15)	Population-based testing is vital in ensuring the containment of the virus.
Jones and Comfort (16)	Medical pressures like availing sufficient testing centers prevailed at the pandemic's beginning.

## Discussion

### Summary of Evidence

#### Creation of Jobs in the Pharmaceutical Industry

The pandemic has presented opportunities for more employment chances in other sectors like pharmacy. According to a study by Miglierini, the requirement to transfer some pharmacy active ingredients and production to Western states may offer more long-term employment chances (3). The basis of this argument is that many crises, such as World War 1 and influenza, often experience strong economic growth in the end. Another article by Morriss expounds on this point by stating that the number of applicants to join the pharma world has increased since lockdown (4). Therefore, there is a likelihood that more people will get jobs to replace the healthcare workers that succumbed to the virus. The hiring of more professionals will be necessary, ultimately raising the talent bar in the field. Such a situation will present more chances for employment. A different document by Singh reveals that the pandemic has increased the demand for medicines globally (5). This demand is likely to increase since the pandemic is not yet over. Hence, this instance implies that more employees are needed to distribute the medicine to different people globally.

The COVID-19 pandemic revealed that the world has an insufficient workforce to handle all the challenges brought about by the virus. An article by Akande-Sholabi and Adebisi reveals that the pandemic revealed the inability of many developing countries to sufficiently cater for themselves, thereby depending on external aid (6). For instance, Nigeria produces all its medication using active ingredients sourced from India and the Peoples Republic of China (6). This situation is risky and requires developing countries to invest in producing medicine locally. Such an instance will prompt the development of the domestic pharmaceutical industry, thereby creating employment for residents. In a similar argument, Ayati, Saiyarsarai, and Nikfar claim that the long-term effects of the COVID-19 pandemic include the pharmaceutical sector establishing self-sufficiency in the industry's production and supply chains (7). This statement implies that many governments strive to ensure that their pharmaceutical industry can easily supply the medicine required by residents without fail. As an outcome, governments will have to employ more workforce to assist in attaining this goal. Therefore, the pandemic has made governments notice the problems in their healthcare systems and will have to dedicate efforts to solve them when dealing with the COVID-19 pandemic.

#### Vaccine Sales

The current COVID-19 pandemic has led to the spiraling of the vaccine market. The reason for this is that after comprehensive research, the medical fraternity has identified vaccination as the only way of managing the virus. Many companies manufacturing the vaccine have benefited from the sales. An analysis carried out by Zeitouny et al. revealed that the demand for vaccines was significantly higher in low and middle-income states compared to high-income states during the first wave of the pandemic (8). These factors imply that the enhanced demand for vaccines increased the need for more enterprises to manufacture the product. Furthermore, the statements highlight the point that developing countries have lower access to essential pharmaceutical resources due to the tendency of their countries to source ingredients for medicine production from foreign countries. Bown and Bollyky stated that enterprises such as Pfizer, Moderna, AstraZeneca, and Johnson & Johnson have been at the forefront of assisting the world in ending the pandemic (9). These companies have produced vaccines that prevent people from experiencing the adverse complications associated with the COVID-19 pandemic. The process of manufacturing medication is distinct from that of making vaccines. Therefore, the enterprises mentioned above have the advantage of having the original ingredients used to produce the vaccines. An article by Oxfam highlighted the sales made by the companies by pointing out that Pfizer, BioNTech, and Moderna acquire more than one thousand dollars in profit per second even though most of the people residing in the poorest countries remain unvaccinated (10). This factor raises concern about whether the companies producing vaccines encourage overall economic sustainability.

#### Job Creation in Vaccination Centers

The only way to manage the COVID-19 pandemic is by receiving a vaccination. The requirement for everyone to get vaccinated has opened many job opportunities. For instance, many people have acquired employment positions that require them to distribute the vaccine to residents. For example, a study conducted by Paudyal et al. revealed that vaccination centers in Europe had integrated pharmacists in the process of developing the vaccination program to enhance the public health benefits of the vaccines (11). Thus, this statement implies that the skills possessed by pharmacists are critical in vaccination centers; thereby, there will be more opportunities for applicants for the position to acquire the job. Also, the article reveals that the currently required uptake of the vaccine is 67%, which implies that governments will have to employ more workforce to ensure that they reach this target (10).

Vaccination centers have also created employment opportunities for healthcare professionals as research conducted by Sahin and Sahin showcased that the vaccination process will present physicians with the chance to identify any other problems associated with the pandemic that patients have (12). Such a situation will trigger recognizing conditions like mental health issues and chronic conditions that a qualified healthcare worker will manage to prevent adverse consequences. As a result, vaccination centers are creating jobs and enabling medical practitioners to identify gaps concerning the type of care patients receive and integrate the necessary remedies. These centers can create additional employment that will require the respective companies to hire a different workforce to deal with the demand for various services. The future of jobs in the vaccination centers is uncertain, as an article by Rubin revealed that vaccine centers may have to come up with annual booster vaccines to deal with newer variants (13).

#### Job Creation in Testing Centers

After the onset of the COVID-19 pandemic, many governments encouraged their citizens to get tested and prevent further spreading of the illness if they found out they had the disease. A study conducted by Flynn et al. revealed that the process of testing individuals for the virus is critical and requires the contribution of healthcare workers (14). This phrase implies that the demand for healthcare workers in the pharmaceutical industry is high, and these numbers will go higher depending on how long the COVID-19 pandemic prevails. Testing centers have created employment opportunities in the medical domain since many states require conducting population-based testing of all individuals, even those without symptoms, to promote the containment of the virus, as pointed out by Fragala, Goldberg, and Goldberg (15). The importance of large-scale testing will drive governments to hire more personnel to guarantee the mass vaccination of most of the population. Other than testing patients, testing centers provide additional job opportunities to evaluate the acquired tests and deliver accurate results. An article by Jones and Comfort revealed that at the beginning of the pandemic, many countries globally faced medical pressures associated with availing enough testing facilities (16). This factor implies that given that the pandemic is still ongoing, states will require to open up more testing facilities and hire more medical staff to run the organizations.

### Limitations

The main limitation of this study is the lack of sufficient information on the topic under study. Since the COVID-19 situation is a new research area, very few researchers have evaluated the factor in job creation in the healthcare domain. This gap implies that the information provided in the manuscript may not be conclusive as it only considers research from a few pieces of literature. Another limitation of this study is that the study limit was 1 year (from 2020 to 2021), which contributed to the few documents acquired since studies on the topic are still underway as it is a new area in the field of research that requires deeper evaluation. Moreover, this study faces the limitation of only using articles published in English. This factor could have contributed to the low number of literature found. There could have been some articles containing relevant information in other languages like Spanish and French. Therefore, the information generated through this study could lead to misleading conclusions since additional information is necessary to ensure the availability of enough supporting evidence.

## Conclusions

In summary, the results of this review indicate that the COVID-19 pandemic has created numerous employment opportunities in the pharmaceutical industry, vaccination, and testing centers during the pandemic time. However, vaccine makers may develop annual boosters to deal with emerging COVID-19 variants. Nonetheless, the necessity of the booster vaccines is uncertain, making this claim inconclusive. The results generated *via* this review are essential as they will contribute to the literature on the topic that is currently minimal. Future researchers can use the outcomes to add on them or criticize them. Also, governments will benefit from these results as they can use them to structure their distinct healthcare policies to ensure that they guarantee maximum benefits to healthcare workers. The recommendations for future research include more researchers evaluating the topic to provide a vast wealth of literature on the subject. Researchers should ensure this by engaging in comprehensive studies that use evidence-based practices to acquire information. Increasing the number of studies in the sector will guide governments and the relevant stakeholders in the pharmaceutical industry with the information they need to increase the number of jobs in the healthcare domain by hiring more staff in vaccination and testing centers. The absence of extensive literature on the topic presently challenges researchers to develop primary research that uses raw data to derive conclusions. This approach will be more reliable than using secondary sources.

## Author Contributions

AT and ST-M: conceptualization. BE: data collection. ST-M: visualization and writing—review and editing. AT and MB: writing—original draft. ST-M and DMF: writing—review and editing. All authors have read and agreed to the published version of the manuscript.

## Conflict of Interest

The authors declare that the research was conducted in the absence of any commercial or financial relationships that could be construed as a potential conflict of interest.

## Publisher's Note

All claims expressed in this article are solely those of the authors and do not necessarily represent those of their affiliated organizations, or those of the publisher, the editors and the reviewers. Any product that may be evaluated in this article, or claim that may be made by its manufacturer, is not guaranteed or endorsed by the publisher.
